# Strychnia in “Hay Fever” and Influenza

**Published:** 1861-03

**Authors:** James Barrach

**Affiliations:** Philadelphia


*Strychnia in "Hay Fever" and Influenza.* By JAMES BARRACH, M. D., of Philadelphia.

Within the last few years it has been my lot to have several persons under my charge who were suffering from the affection known as "hay fever" or "hay asthma."

The first case was that of a lady about 50 years of age, of nervous temperament, who had been afflicted with this malady for seven years previous to my seeing her. She had been under various treatment, but without obtaining alleviation. Her attacks occurred every year—almost to the day—on the 16th of August, and lasted from nine to ten weeks, during which time she was more than miserable. The onset consisted in unpleasant feelings in the nose, sometimes of a tickling nature. These lasted about two days and were soon followed by sneezing, extreme lachrymation and flowing of thin mucus from the nostrils. During the intervals of sneezing and lachrymation, her head, especially in the region of the frontal sinuses, felt much constricted and painful, while the throat, fauces and soft palate itched to a painful degree. At night the constricted feeling about the head would continue, together with a stuffing of the nostrils, which would prevent her breathing by the latter; and, to add to her misery, when she attempted to breathe by the mouth the tickling sensation in the fauces became intolerable.

The above symptoms were accompanied with coldness of the surface, especially of the feet and legs. During the attack her appetite and digestion were good, unless taken away by the use of nauseating and depressing remedies.

While listening to the recital by the above named patient of the phenomena of her disease, my mind was occupied by three prominent ideas in relation to the malady: 1st, its periodicity; 2d, its prolonged course; and 3d, that it was based on debility, and that this debility was owing to some mal condition of the nervous system, which manifested itself in relaxed and weakened capillaries. Its periodicity suggested some anti-periodic; its prolonged course, that if the disease could not be cut short that at least depressing and relaxing remedies would most likely be injurious, however useful such remedies might be found in other acute affections of the respiratory mucous membrane; while the view which had been taken as to the condition of the capillaries, based on a debilitated nervous system, suggested the use of strychnia. That this remedy should exert a beneficial influence in the case seemed probable to me from the happy effects of it in the treatment of passive forms of dysentery and diarrhœa.—Whether right or wrong in the above views, the result was most satisfactory, and also pleasing, that it was the application of principles, rather than empiricism which suggested the remedy. The strychnia was administered in this case in doses at first of gr. one-thirtieth, and afterwards gr. one-twentieth, four times daily, and with most manifest relief, especially to the more decided influenza symptoms in the affection of the mucous membrane.

In addition to the above treatment the skin was stimulated by hot salt baths and frictions, and the extreme tickling of the fauces and soft palate was alleviated by a gargle of sulphate of zinc gr. v. to the ℥ i of water. The stuffing of the nostrils at night was relieved when necessary by a hot toddy. I attempted to cut short the disease by quinine, administering it for a few days before the expected attack, but without success. Fowler's solution was also used, and likewise failed.

This lady was under my care for four successive summers, and each season her attack was shortened from ten to five and three weeks, until, finally, the last season was passed with almost entire immunity from the enemy.

The second case which came under my charge was that of a lady about 33 years of age, also of extremely nervous temperament, who had suffered with the disease for the most of her life, and whose symptoms were much as mentioned in the above case, with the additional ones of almost constant lancinating pains through the eyes, with daily attacks of agonizing itching of the conjunctivæ.

The first of these symptoms was relieved by belladonna, and the latter by dropping in the eye a weak solution of salt and water. The strychnia gave most prompt relief to the influenza symptoms.

The third case was that of a man æt. 40 years, who had had the complaint all his life, and had been under respectable practitioners, and his report was that he despaired of relief. During the past summer he came under my charge but neglected to place himself under treatment until he had been three days suffering from the attack. The symptoms were those of extreme influenza. He also told me that his bronchial tube soon became affected, which complication was not an attendant of the first two cases. He was immediately placed under the use of the strychnia, in the form of the sulphate, gr. one-sixteenth, four times daily, and in two days he was relieved—I may say cured—for the season.

A fourth case of this disease was treated by this remedy by another physician, in accordance with my advice, and the report was very favorable. I heard from the patient but once.

Since treating the above cases, I have given the strychnia in ordinary influenza in the case of a distinguished friend—a physician. On one occasion he mentioned to me that he was getting one of his attacks of cold, which usually lasted about three weeks, affecting his mucous membrane generally—bronchia, stomach and bowels—and usually ending with diarrhœa.

I found him sneezing, and his eyes and nose discharging heavily, and his skin realxed, moist and cool. These symptoms, I thought, eminently indicated the above remedy, and by my advice he took gr. one-twentieth, four times daily, and in two days was perfectly well. There was no return.

In connection with the above treatment, although it is not my intention to discuss the pathology of "hay asthma," much less that of influenza in general, I would add that living in the country always aggravated the symptoms to a great degree; also, as is generally understood, the smelling of flowers, and more especially the dust from hay, or the patient moving about where any fine particles are abundantly disseminated in the air.

The sea-shore life, which is considered beneficial to these cases, did not prevent one of my patients from having her usual attack, although by anticipating the time and gaining the shore before the attack, it seemed to have been retarded about a week; but while walking on the beach, against a strong cold wind, she felt her forehead over the frontal sinuses become cold, and soon after the disease came on in full blast.

Since the idea of the use of strychnia in this complaint first occurred to me I have discovered in the *London Lancet*, Vol. 1, for 1850, the mention of the successful use of nux vomica, by Dr. Greaves, in hay fever. Among the remedies which are highly recommended in this disease are tincture of lobelia and coffee.—*Maryland and Virginia Medical Journal.*

